# Integration of Electrodeposited Ni-Fe in MEMS with Low-Temperature Deposition and Etch Processes

**DOI:** 10.3390/ma10030323

**Published:** 2017-03-22

**Authors:** Giuseppe Schiavone, Jeremy Murray, Richard Perry, Andrew R. Mount, Marc P. Y. Desmulliez, Anthony J. Walton

**Affiliations:** 1 SMC, School of Engineering, University of Edinburgh, Edinburgh EH9 3FF, UK; jeremy.murray@pyreos.com (J.M.); anthony.walton@ed.ac.uk (A.J.W.); 2 School of Chemistry, University of Edinburgh, Edinburgh EH9 3FF, UK; richard.perry@ed.ac.uk (R.P.); a.mount@ed.ac.uk (A.R.M.); 3 School of Engineering & Physical Sciences, Heriot-Watt University, Edinburgh EH14 4AS, UK; m.desmulliez@hw.ac.uk

**Keywords:** Ni-Fe integration, Permalloy, MEMS, surface micromachining, selective etching, Ni-Fe electroplating, magnetic microactuators

## Abstract

This article presents a set of low-temperature deposition and etching processes for the integration of electrochemically deposited Ni-Fe alloys in complex magnetic microelectromechanical systems, as Ni-Fe is known to suffer from detrimental stress development when subjected to excessive thermal loads. A selective etch process is reported which enables the copper seed layer used for electrodeposition to be removed while preserving the integrity of Ni-Fe. In addition, a low temperature deposition and surface micromachining process is presented in which silicon dioxide and silicon nitride are used, respectively, as sacrificial material and structural dielectric. The sacrificial layer can be patterned and removed by wet buffered oxide etch or vapour HF etching. The reported methods limit the thermal budget and minimise the stress development in Ni-Fe. This combination of techniques represents an advance towards the reliable integration of Ni-Fe components in complex surface micromachined magnetic MEMS.

## 1. Introduction

Electrochemically deposited alloys of nickel and iron (ECD Ni-Fe) are attractive materials for the fabrication of integrated magnetic microelectromechanical systems (MEMS) [1,2,3,4]. Although highly desirable for their large magnetic permeability and low coercivity [5,6,7,8], ECD Ni-Fe alloys are known to suffer from chemical deterioration when exposed to subsequent processing steps [9] and require therefore careful planning during the process integration stage.

Electroplating requires the preliminary deposition of a conductive seed layer that serves as a nucleation surface for the growth of the desired material through patterned moulds. Copper is one of the most commonly used seed layer materials, as it offers a balance between high conductivity, thus reducing non-uniformities caused by radial voltage drops on the wafer during electrodeposition, and ease of processing [10,11]. After electrodeposition, the mould is stripped and the exposed seed layer must be removed to electrically isolate the individual electroplated structures. To this end, wet etch processes are preferred to dry etching as the latter require expensive tools and possibly further masking. Conventional copper wet etchants are solutions based on (NH_4_)_2_S_2_O_8_, HNO_3_, HCl/CuCl_2_ or HCl/FeCl_3_. None of these chemicals is, however, selective enough to enable the copper to be removed while leaving Ni-Fe intact [12,13]. A method to selectively wet etch copper in the presence of structural ECD Ni-Fe elements using conventional etchants is therefore desirable to facilitate the integration of soft magnetic elements in complex process flows while still employing standard techniques.

Moreover, when manufacturing MEMS devices with mechanically movable components such as microswitches and microactuators, surface micromachining is a key process that enables suspended and freestanding structures to be patterned and released [14]. To integrate moveable soft magnetic components based on ECD Ni-Fe in MEMS devices, it is necessary to develop robust sacrificial etch processes that preserve the integrity of the magnetic and other surrounding materials [15,16,17,18,19]. The release of freestanding structures in MEMS is usually the last step in the manufacturing process flow before packaging, as suspended elements make the devices significantly more sensitive to failure when subject to further processing [20]. For this reason, surface micromachining processes are often performed on complete devices that already incorporate all functional elements. It is therefore crucial to ensure sufficient etch selectivity between the sacrificial material of choice and the combination of other structural materials employed as functional device elements. Additionally, complex architectures require the patterning of sacrificial layers prior to surface micromachining, in order to allow for further processing before the releasing step. Both the patterning and complete removal of sacrificial layers must therefore be fully compatible with Ni-Fe and the other structural materials.

Another key aspect that must be considered when integrating ECD Ni-Fe elements in MEMS process flows is the control of stress and stress gradients, as significant stress development is known to occur in Ni-Fe films during processing, especially when subject to high thermal loads [21,22,23]. Our group has previously reported on the increased levels of stress in ECD Ni-Fe when excessive heat is applied during processing [24,25,26], and identified the thermal budget as a critical obstacle to the integration of the alloys in MEMS devices. Constraints on the processing temperatures and times are therefore introduced, as excessive thermal loads can generate deteriorating levels of residual stress and stress gradient that may impair the functionality and integrity of the microfabricated devices [24,27].

The work reported herein addresses these fundamental challenges associated with the integration of ECD Ni-Fe in complex MEMS process flows. The article reports both a wet etching process to selectively remove the electroplating copper seed layer used as nucleation base for Ni-Fe, and a series of deposition and etching techniques that enable the patterning and release of freestanding Ni-Fe structures surrounded by silicon nitride structural dielectric, using silicon oxide as a sacrificial material that can be first patterned and then completely removed. All the processes developed in this study are run at temperatures below 200 °C, a limit defined based on previous work [25] to prevent excessive development of stress and stress gradients.

The ensemble of the methods presented herein provides a toolkit that aims at facilitating the integration of ECD Ni-Fe in complex MEMS architectures by using selective and low temperature deposition and etch processes.

The paper is organised as follows. Section 2 of the article presents an improved wet etch solution based on ammonium persulfate ((NH_4_)_2_S_2_O_8_) that enables the selective removal of copper seed layers in the presence of electrodeposited Ni-Fe structures. Rotating pointer arm test structures are used to visually monitor the effects and completion of the etch process. The design and fabrication of the test structures are presented first, followed by the experimental details for the etching tests.

Section 3 of the article presents a short discussion on the choice of structural and sacrificial dielectric materials for surface micromachining. The development of low temperature deposition processes for structural silicon nitride and sacrificial silicon oxide films is then presented, followed by the details of a wet etch process based on Buffered Oxide Etch (BOE). This enables the patterning of relatively thick sacrificial silicon oxide (~2 µm) while preserving the integrity of structural silicon nitride dielectric elements.

Section 4 of the article completes the toolkit by presenting a complete surface micromachining process for the manufacturing of freestanding ECD Ni-Fe structures in the presence of silicon nitride as structural dielectric. The proposed method uses the low temperature silicon dioxide sacrificial material and silicon nitride structural material described in Section 3. The sacrificial layer is patterned by wet etching first, and subsequently completely removed by means of a vapour hydrofluoric (HF) acid etch to avoid stiction. All the processes are limited to a maximum temperature of 200 °C, in order to minimise stress generation in Ni-Fe structures.

Finally, Section 5 closes the article by summing up the findings and providing some perspectives on the application of the results achieved.

## 2. Selective Copper Seed Etch

Test structures were produced on 3” silicon wafers to study the effects of different copper etching chemistries and tune the etching process for minimal Ni-Fe attack.

### 2.1. Test Structures

**Figure 1:**
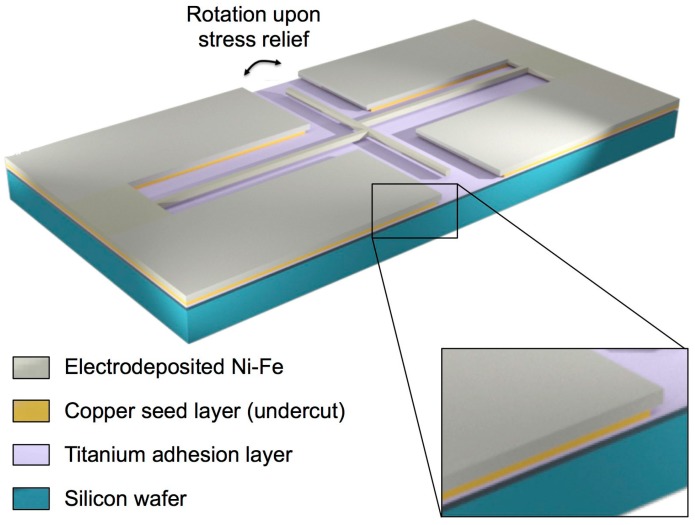
3D diagram of the pointer arm microstructure used to characterise the copper seed layer etch.

A conceptual drawing of the test structures used for this investigation is shown in Figure 1. Rotating structures of this kind are typically used as strain sensors [24,26,27], but they can also indicate the completion of the etch release process.

The structure is fabricated by growing Ni-Fe through a patterned mould and comprises three elements: two parallel expansion arms and a transversal pointer arm. The two expansion arms (8 µm wide, 850 µm long and ~2 µm thick) are attached at one end to a large anchor pad, with the other end connected to the transversal pointer arm with an offset joint.

### 2.2. Fabrication

**Figure 2:**
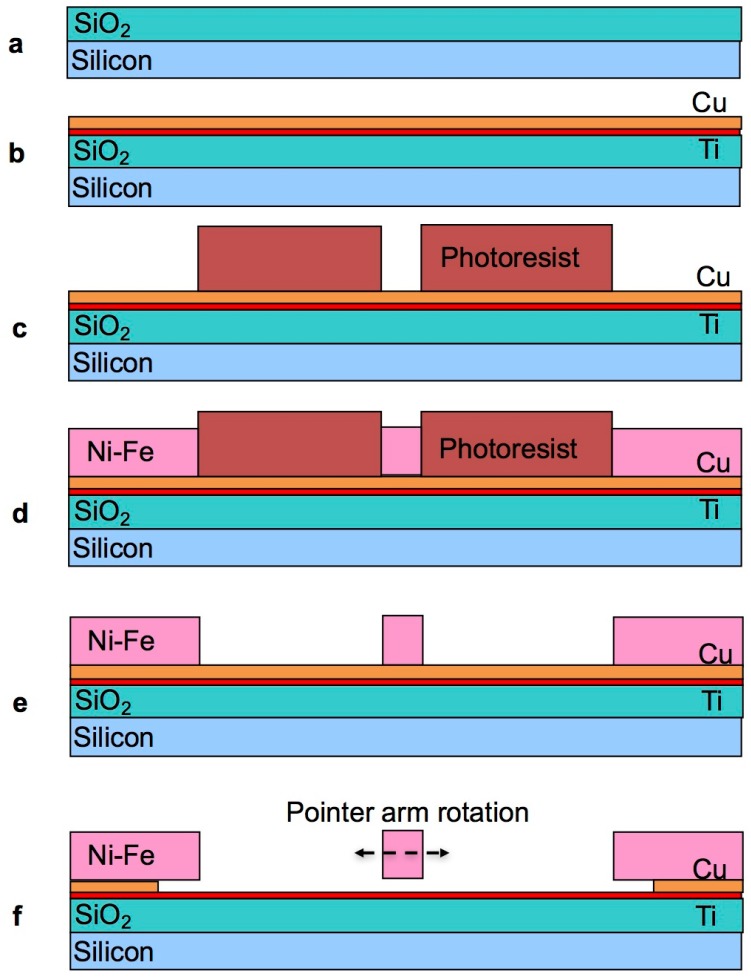
Fabrication process flow for the rotating pointer arm test structures: starting silicon substrate with protective silicon dioxide coating (**a**); sputter deposition of the titanium-copper seed layer stack (**b**); lithography to define the electroplated areas (**c**); electrodeposition of Ni-Fe through the photoresist mould (**d**); photoresist stripping and exposure of the copper seed layer (**e**); etching of the exposed copper seed layer and undercut etch underneath the pointer arm structures (**f**).

The fabrication of the test devices follows the diagram illustrated in Figure 2.

The starting substrate is a silicon wafer coated with a protective layer of 0.7 µm thick silicon dioxide (Figure 2a). The titanium and copper seed layer stack is then sputter deposited on the wafer in an OPT Plasmalab 400 magnetron sputtering system (Figure 2b). The copper serves as a seed layer for electroplating at a thickness of 300 nm to ensure good conductivity over the entire wafer area. The 30 nm thick titanium serves as adhesion layer between the copper and the underlying silicon dioxide insulator. Photolithography is then used to pattern the electroplating mould (Figure 2c). To this end, the wafer is first exposed to adhesion promoter hexamethyldisilazane (HMDS) at room temperature for 30 s, then Microchem MEGAPOSIT™ SPR™ 220-4.5 photoresist is dispensed and spun at 4000 rpm for 60 s, so as to obtain a thickness of about 4 µm. An edge bead removal (EBR) step is performed to ensure that the seed layer is exposed at edge of the wafer, as it will serve as contact for the electrodeposition process. The photoresist is then soft baked at 90 °C for 60 s. The wafer is placed in the mask aligner, operated in proximity mode, and exposed to a dose of about 350 mJ/cm^2^. The photoresist is post-exposure baked at 115 °C for 90 s, and then developed in MEGAPOSIT™ MF26A developer until all the exposed photoresist is dissolved. A ~2 µm thick Ni-Fe layer is then electrodeposited using a DC power source to provide a current density of 20 mA/cm^2^ (Figure 2d). The electroplating bath is composed of the chemicals reported in Table 1.

**Figure 3:**
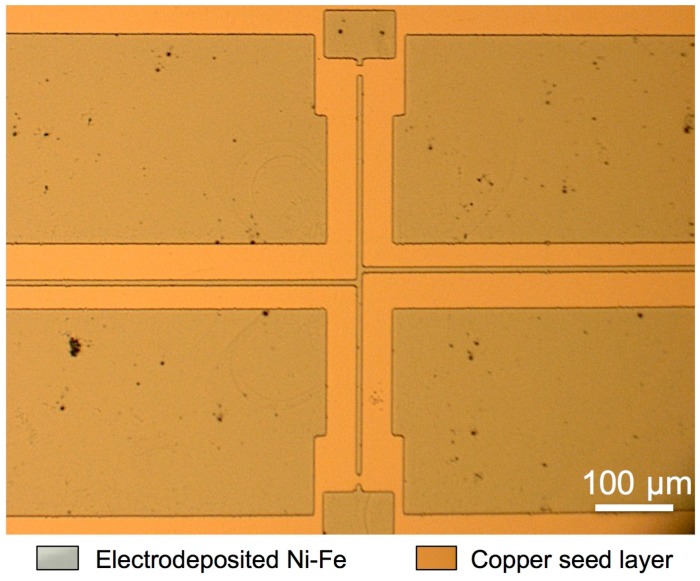
Micrograph of Ni-Fe electrodeposited test structure anchored to the copper seed layer.

The thickness (2 ± 0.2 µm) and alloy composition (Fe contents of around 20% ± 5%) of the films were verified by means of X-ray fluorescence measurements. Once the electrodeposition is completed, the photoresist is stripped to expose the copper seed layer for wet etching (Figure 2e). Figure 3 shows a photograph of a test structure at this point in the process.

At this stage, the copper seed layer is etched (Figure 2f). The exposed copper surface is etched first, followed by the undercut underneath the Ni-Fe structures. Once the undercut etch is sufficient to completely remove the copper underneath the narrow beams (4 µm sideways etch), these are no longer bound to the substrate and free to contract or expand to relieve the residual stress. The observation of a movement of the pointer arm is therefore an indication of the completion of the copper etch underneath the beams. The larger electrodeposited areas of the anchor pads at this stage remain fully anchored to the substrate, as the undercut only extends to half the width (4 µm) of the narrow Ni-Fe beams, plus any over-etch.

### 2.3. Development of the Selective Etching Process

Permalloy (80:20) and nickel-iron alloys in other ratios are reportedly attacked by ferric chloride etchants [28,29,30], cupric chloride etchants [12,31] (although they might not attack pure nickel [32], their oxidising action attacks the iron component [31]), HNO_3_, HCl, H_2_SO_4_, H_3_PO_4_ and HF [33]. In this work, the copper layer used as electrodeposition seed is etched with ammonium persulfate. To check the compatibility of this chemistry with ECD Ni-Fe, a first test was conducted by immersing test structures anchored to the underlying copper seed layer in a solution of ammonium persulfate diluted to a concentration of 20 gL^−1^ in deionised (DI) water. The etching reaction of copper in ammonium persulfate is

Cu_(s)_ + S_2_O_8_^2−^ ⟶ Cu^2+^ + 2SO_4_^2−^ (1)

**Figure 4:**
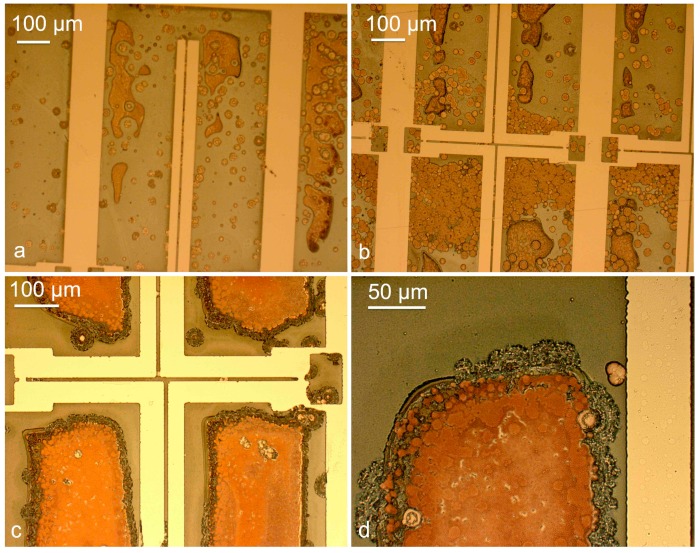
Micrographs of the test structures subject to copper etch with ammonium persulfate, at different etch times: 3 min (**a**); 5 min (**b**); and 10 min (**c**,**d**).

Three test wafers were left in the solution for 3, 5, and 10 min to monitor the progressive effects of the etchant on the ECD Ni-Fe. Figure 4 illustrates the results.

The micrographs of Figure 4 reveal two different undesired phenomena, namely the chemical attack of Ni-Fe, more evident in Figure 4c, and the redeposition of the etched copper on the anchored surfaces, even at the first stages of the etch (Figure 4a). Figure 4d shows in detail the redeposited copper layer on the ECD Ni-Fe. After 10 min in the etching solution the beams appear to be still anchored, suggesting that copper residue is still present underneath the narrow structures, while the edges of the ECD Ni-Fe structure exhibit signs of corrosion.

This highlights a problematic lack of selectivity. Wu et al. have shown that a pH in the alkaline range can help mitigate this phenomenon [9]. In light of this, ammonium persulfate was diluted this time to a concentration of 20 gL^−1^ in 1 M sodium hydroxide (NaOH, 40 gL^−1^), in order to characterise the etching reaction at a higher pH (~12). A progressive pH decrease is expected during the etch in this solution as the OH^−^ groups from the sodium hydroxide react to form water:

Cu^2+^ + 4NH_4_^+^ + 4OH^−^ ⟶ Cu(NH_3_)_4_^2+^ + 4H_2_O. (2)

This is more evident when small volumes of the etching solution are prepared.

**Figure 5:**
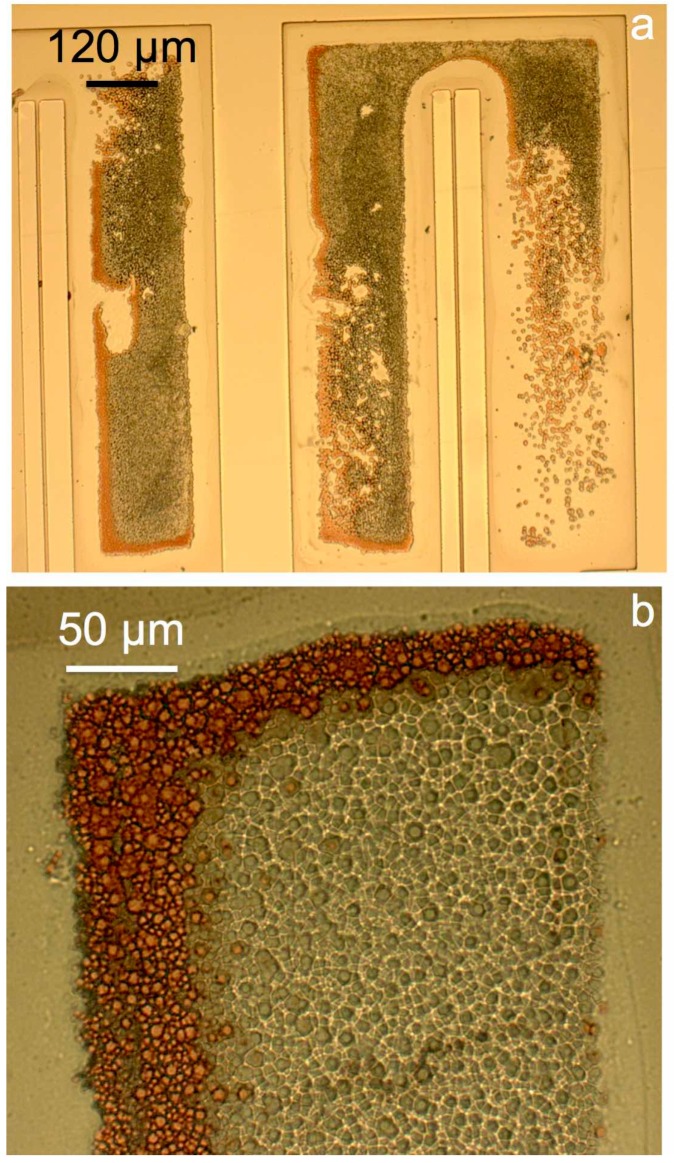
Micrographs of samples used for copper etch tests with ammonium persulfate in 1 M NaOH: overview of an anchor pad (**a**) and detail of the surface (**b**).

A test wafer with the same test structures was immersed in the high pH etching solution for 10 min, and the results are shown in Figure 5.

The images show satisfactory selectivity, with the Ni-Fe structures exhibiting no visible signs of corrosion (at this scale and compared to Figure 4c) after 10 min in the etching solution. However, the copper seed is still present underneath the electrodeposited structures after this relatively long etch time, as indicated by the narrow beams in Figure 5a being straight and anchored. To increase the etch rate, the concentration of ammonium persulfate in the sodium hydroxide solution was increased to 50 gL^−1^.

As for the redeposition of etched metal observed prevalently on the bigger areas of the ECD structures, this effect has been reported in the literature and solved by adding a variety of complexing agents to the etching solution, such as 1,4,8,11 tetraazundecane [34] or dimethyl sulfoxide [35]. In this work, a different complexing agent is chosen, citric acid (C_6_H_8_O_7_), as it has the advantages of being inexpensive and commonly available. The addition of citric acid causes the formation of copper citrate complex and ammonia gas, according to the reaction

Cu(NH_3_)_4_^2+^ + {Cit}^3−^ ⟶ Cu{Cit}^−^ + 4NH_3(aq)_, (3)

where {Cit}^3−^ is the deprotonated species (C_6_H_5_O_7_)^3−^ resulting from dissociation of citric acid in a high pH solution.

**Figure 6:**
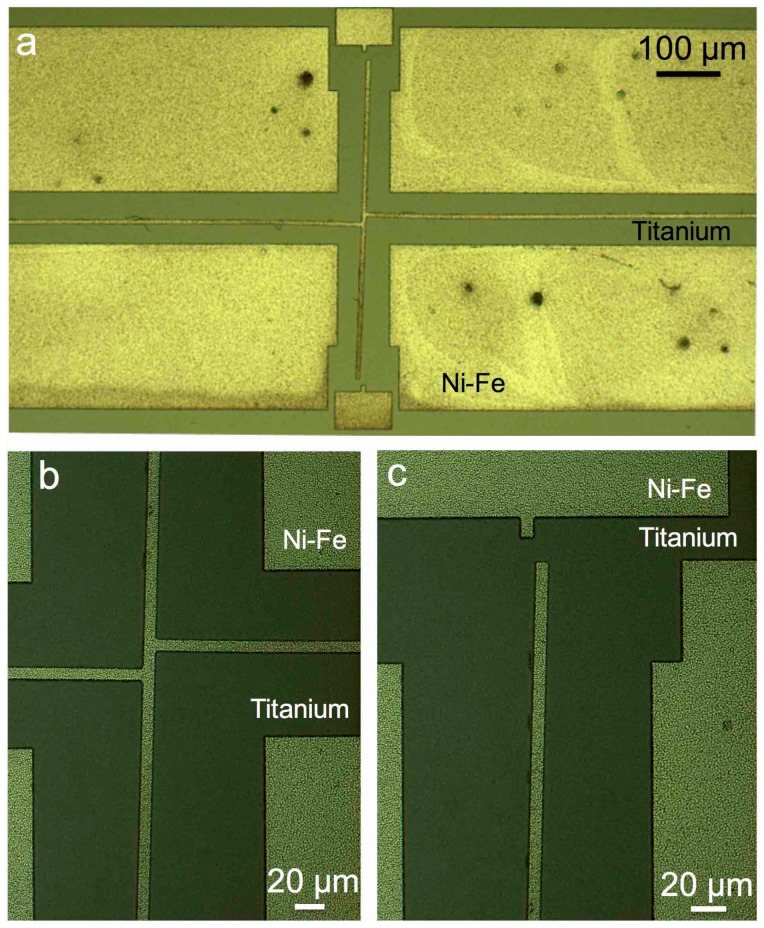
Photographs of ECD Ni-Fe structures successfully released from the copper seed layer after 4 min in ammonium persulfate diluted in 1 M NaOH and complexing agent: (**a**) overview of a test structure, and details of the offset joint in the centre (**b**); and pointer arm tip (**c**). Micrographs (**b**) and (**c**) are taken from a different structure. Note the titanium adhesion layer is visible below the ECD Ni-Fe after the copper is removed.

To verify the effectiveness as complexing agent, 50 gL^−1^ of citric acid were dissolved in the etchant to prevent the reduction of copper on Ni-Fe. The new etching solution was tested on another wafer and the results are presented in Figure 6. The undesired effects of Ni-Fe corrosion and redeposition of etched metal are eliminated and the copper seed layer is completely removed after 3–5 min in the etchant, as indicated by the rotation of the pointer arms and the clean wafer surface, which appears free of residue.

A series of etching solutions was then tested with ammonium persulfate concentrations ranging from 10 gL^−1^ to 50 gL^−1^. The observed effect on the copper seed etching process is that higher concentrations of ammonium persulfate offer higher etch rate, without affecting the other exposed materials. If the solution is prepared in small volumes, a slight increase in the etch time for 300 nm sputtered copper is observed when large numbers of wafers are processed. No increase in the etch time was observed when etching up to a total of four patterned 3” silicon wafers in 200 mL of etching solution. Table 2 summarises the composition of the final developed etching solution.

This proposed solution enables the release of the presented pointer arm test structures by etching 300 nm thick copper with an undercut of 4 µm in 3–5 min on average. This etch is a two-stage process which starts by removing the visible copper and consequently exposing the top part of the copper underneath the Ni-Fe. The etch process continues sideways underneath the Ni-Fe structures once the whole thickness of the copper is etched in the uncovered areas. This etch process causes no visible attack on Ni-Fe and titanium even after exposure for 10 min to the solution. This result opens wider possibilities of using ammonium sulphate based copper etchants in the presence of nickel and iron alloys, where other chemistries do not provide sufficient selectivity.

## 3. Selective Sacrificial Layer Etching

A critical requirement for surface micromachining is the choice of a suitable sacrificial layer. Desirable properties sought in the sacrificial material include robustness to support ECD Ni-Fe structures through other fabrication steps prior to the release, ease of etching for both patterning and final removal, and etch selectivity with respect to other structural materials.

### 3.1. Sacrificial Material

Common materials used as sacrificial layers in surface micromachined MEMS are amorphous silicon [36,37], polysilicon [37,38,39], polymers such as polyimide [20,40,41] and Parylene [42], and PECVD silicon dioxide [43]. In the case of complex devices with suspended ECD Ni-Fe structures, the authors have shown that surface micromachining processes employing polysilicon or polymers as sacrificial layer may cause a progressive development of stress gradient in the electrodeposits, due to oxidation of the Ni-Fe film and the thermal budget applied during the required fabrication steps [25]. Silicon dioxide, on the other hand, can be deposited using a large variety of processes, producing films that exhibit different chemical and mechanical properties. Although SiO_2_ can be selectively etched in HF-based chemistries with respect to silicon and some common metals, the etch rates of silicon nitride in such etchants can exhibit significant variation depending on the deposition processes, with low and high selectivity reported [43]. In particular, silicon nitride deposited by Low Pressure Chemical Vapour Deposition (LPCVD) shows good selectivity against HF-based SiO_2_ etch [44], but it requires very high deposition temperatures (above 700 °C).

This and similar high-temperature processes are excluded in this work to ensure compatibility with MEMS architectures where Ni-Fe elements are already present on the wafer at earlier fabrication stages (e.g., embedded magnetic cores).

To this end, a process is proposed herein that enables the wet etch patterning of sacrificial SiO_2_ whilst using low-temperature Plasma Enhanced Chemical Vapour Deposition (PECVD) silicon nitride layers as a structural dielectric for complex devices. To enhance the wet-etch selectivity between sacrificial silicon dioxide and structural silicon nitride, a low temperature film is used as sacrificial layer. This has been achieved with a Surface Technology Systems (STS) Multiplex PECVD tool by lowering the temperatures of both the wafer holder and the gas inlet unit in the plasma chamber to 120 °C and by dropping the process frequency from the standard RF value of 13.56 MHz to 380 kHz. Tuning the properties of PECVD deposited dielectric layers by modifying the process conditions is a technique that has been widely adopted for a range of applications. The influence of dual high and low frequency PECVD parameters and substrate temperature has been used for instance to control the residual stress and mechanical properties in silicon nitride [45,46] films. On the other hand, low-temperature PECVD silicon oxides have been investigated for Through Silicon Via applications, benefitting from satisfactory step coverage and electrical properties while easing the process integration by avoiding excessive thermal loads [47,48]. In this work, a combination of low frequency and low temperature is used for the PECVD deposition of sacrificial silicon oxide. The low temperature enables the deposition of low density oxide that can benefit from a higher etch rate [49], while the low frequency introduces an ion bombardment mechanism that enhances chemical reactions and causes low energy ion implantation [50], improving the stability of the film when subjected to further processing [51].

To test the newly designed sacrificial material, blanket layers of ~2 µm thick silicon dioxide were produced using the PECVD process parameters listed in Table 3. The thickness of 2 µm was chosen to emulate typical vertical gap dimensions for MEMS actuators [52].

In addition, blanket silicon nitride structural layers were also PECVD deposited to enable etch rate comparisons. The process parameters used for the deposition of 200 nm Si_3_N_4_ are detailed in Table 4. Note that the temperature for this process is also maintained below the limit of 200 °C observed for this study.

### 3.2. Selective Wet Etching of Sacrificial SiO_2_

**Figure 7:**
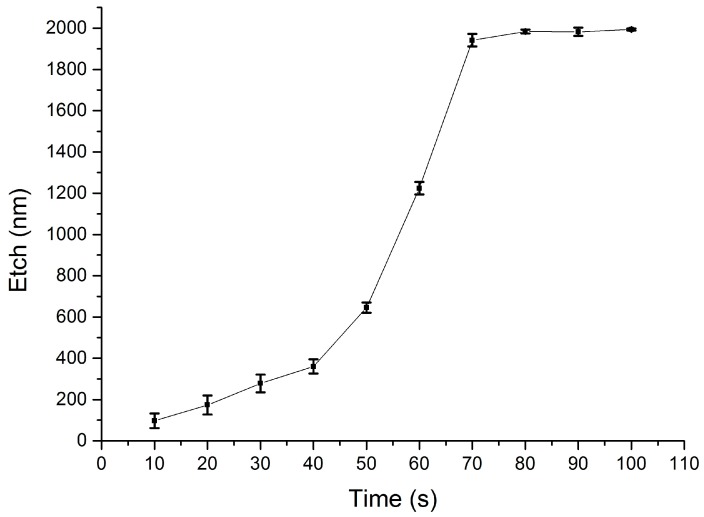
Etched thickness of sacrificial PECVD SiO_2_ in a solution of 1 part BOE 10:1 diluted in two parts DI water. Each measurement point is the average of five thickness measurements across the wafer surface.

The etch rate of the low-temperature SiO_2_ films in BOE 10:1 (10:1 ammonium fluoride (NH_4_F) and hydrofluoric acid (HF)) at room temperature was tested first. To this end, a solution of 100 mL BOE 10:1 diluted in 200 mL DI water was prepared. Test wafers coated with the blanket sacrificial SiO_2_ layer were immersed and left in the etchant for different times, with the etch duration increased in steps of 10 s. The residual film thickness was measured with a Nanospec 3000 reflectometer on each sample, and the results are shown in Figure 7.

**Figure 8:**
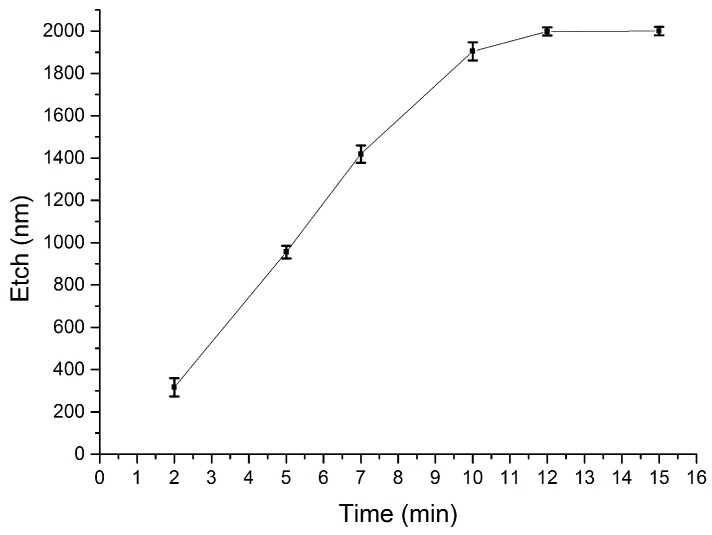
Etched thickness of sacrificial PECVD SiO_2_ in a solution of one part BOE 10:1 diluted in 10 parts DI water. Each measurement point is the average of five thickness measurements across the wafer surface.

These measurements confirm a fast etch rate that removes the entire thickness of the sacrificial SiO_2_ in less than 80 s. To monitor the controllability of the etching process, a further diluted solution was prepared by mixing 1 part of 10:1 BOE in 10 parts of DI water. The experiment was repeated on test wafers identical to the ones used for the previous etch measurements, and the resulting removal curve is shown in Figure 8.

The etch rate of sacrificial SiO_2_ in the 1:10 diluted solution is much more controllable and enables the removal of the entire thickness of 2 µm in about 12 min. The dilution level of BOE can therefore be increased to obtain a controllable etch, or reduced to accelerate the process.

**Figure 9:**
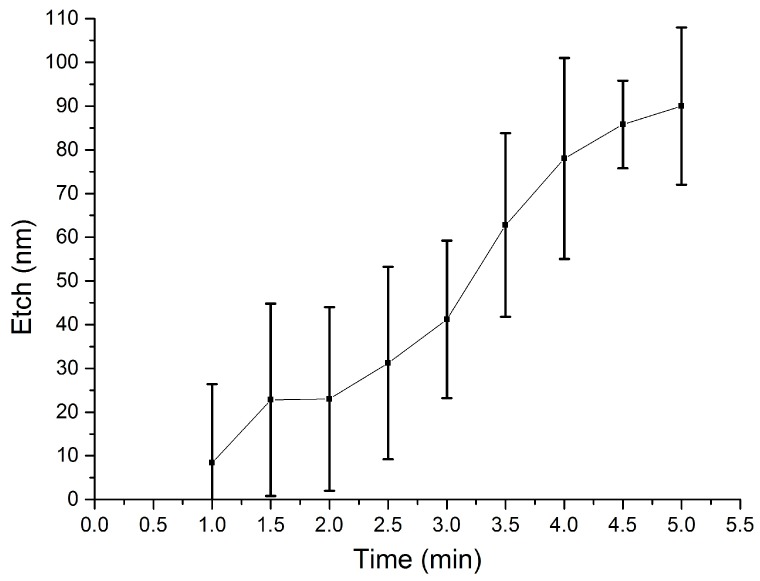
Etched thickness of the structural silicon nitride in a solution of one part BOE 10:1 diluted in two parts DI water. Each measurement point is the average of five thickness measurements across the wafer surface. Note the magnitude of the dispersion bars, as the thickness range is much lower.

To assess selectivity with respect to structural dielectrics, the etch rate of structural PECVD Si_3_N_4_ was measured in the more concentrated BOE solution. The experiment is performed analogously to the etching tests described above, and Figure 9 shows the resulting curve. The silicon nitride exhibits a fairly slow rate of removal, with the thickness of the structural dielectric being reduced by a maximum of 50 nm after the material is exposed to the etchant for 2 min. Figure 7 shows that during this time the same etching solution can remove 2 µm of sacrificial silicon dioxide.

When the experiment on structural silicon nitride was repeated in the 1:10 diluted BOE solution, no appreciable change in thickness was measured for 10 min. These results demonstrate the feasibility of patterning sacrificial structures by wet etching low temperature, low frequency PECVD SiO_2_ whilst preserving the integrity of exposed PECVD Si_3_N_4_. The silicon nitride can therefore be employed as a protective or structural dielectric in complex devices. Approximate values for the etch rates are calculated and detailed in Table 5.

The deposited sacrificial silicon dioxide films exhibit faster etch rates in BOE compared to films produced at standard conditions [34] (for effective comparison, note the further dilution of BOE in this study), and both at RF and low frequency [51].

The calculated selectivity between PECVD SiO_2_ and PECVD Si_3_N_4_ in the more concentrated wet BOE chemistry is therefore around 75. Although far from the very high values of around 850 reported for phosphosilicate glass (PSG) against Si-rich LPCVD silicon nitride [34], the methods proposed in this work trade off selectivity in favour of compatibility with Ni-Fe elements, by using low-temperature deposition processes. This is a crucial aspect for the reliable integration of Ni-Fe in complex MEMS devices such as magnetic actuators, where buried magnetic cores and vias are required prior to the surface micromachining steps to release the actuator elements.

## 4. Surface Micromachining with Vapour HF Etch

In this final section, a vapour phase HF etch is added to the set of viable processes that can be used with the combination of materials studied. Surface micromachining is demonstrated employing the wet etch processes described in the previous sections, plus a vapour phase HF release etch. To this end, microcantilever test structures were fabricated on 3” silicon wafers using ECD Ni-Fe (Table 1) and PECVD silicon nitride (Table 4) as structural materials, and PECVD silicon dioxide (Table 3) as sacrificial layer. This configuration was chosen as it mimics the manufacturing complexities and limitations encountered when producing magnetic MEMS devices such as microactuators.

### 4.1. Test Structures

Test microcantilevers (200–300 µm long and 10–100 µm wide) were manufactured using low-stress electrodeposited Ni-Fe [24,27,53]. A two-step electrodeposition technique was used to first deposit the cantilever anchor, followed by the cantilever beam. This two-stage process avoids the introduction of additional mechanical stress and stress gradients in ECD Ni-Fe [25], enabling a clearer evaluation of the etch release. The sacrificial PECVD silicon dioxide reported in Section 3.1 was used to produce mesa structures that serve as a resting surface for the cantilever beams.

**Figure 10:**
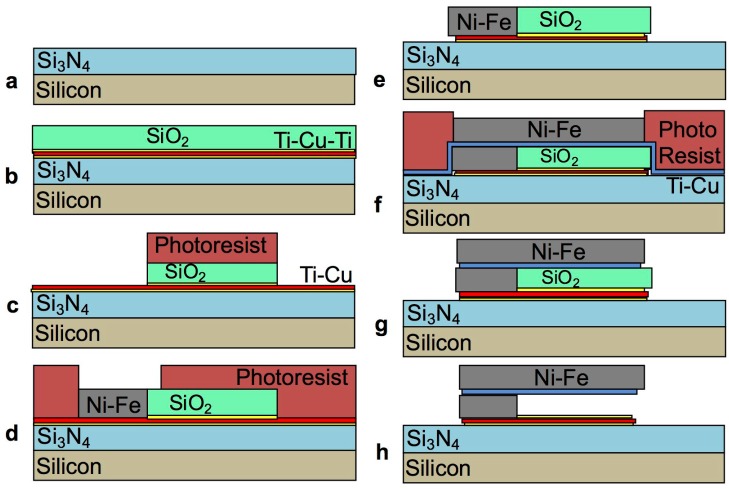
Cross-sections of the process flow for the manufacture of the cantilever test structures: starting silicon substrate with structural silicon nitride layer (**a**); sputter deposition of a titanium-copper-titanium seed layer stack and PECVD deposition of the sacrificial silicon oxide (**b**); lithography and wet-etch patterning of the sacrificial silicon dioxide and the topmost titanium protective layer (**c**); lithography and electrodeposition of the Ni-Fe anchors through the photoresist and silicon dioxide mould (**d**); photoresist strip and wet etch of the copper and titanium seed layer (**e**); sputter deposition of a titanium-copper seed layer stack, lithography to define the cantilever beams, and electrodeposition of Ni-Fe cantilever beam through the photoresist mould (**f**); photoresist strip and wet-etch of the exposed titanium-copper seed layer (**g**); complete removal of the sacrificial silicon dioxide and release of freestanding cantilevers (**h**).

With reference to Figure 10, the process flow comprises of the following steps: (a) PECVD deposition of 200 nm structural Si_3_N_4_ on a silicon substrate as per Table 4; (b) Sputter deposition of 30 nm Ti, 300 nm Cu, 30 nm Ti seed layer stack for electrodeposition. The topmost Ti layer is added to the seed layer stack both to protect the underlying copper from oxidation prior to the electrodeposition step, and to promote the adhesion of the subsequent sacrificial silicon oxide layer, PECVD deposited to a thickness of 2 μm as per Table 3; (c) Photolithography and wet etch of the sacrificial layer in diluted BOE (Table 5) to define the mesa structures that support the cantilever beams, followed by a brief titanium wet etch in 1% HF to expose the conductive copper seed layer; (d) Photolithography to define the cantilever anchors and electrodeposition of Ni-Fe (Table 1) to a thickness that matches the sacrificial mesa; (e) Photoresist strip and wet etch of the Ti-Cu seed layer as per Section 2.3; (f) Sputter deposition of 30 nm Ti and 300 nm Cu seed layer, photolithography to define the cantilever beams and electrodeposition of 2 µm Ni-Fe as per Table 1; (g) Photoresist strip and wet etch of the Ti-Cu seed layer as per Section 2.3; (h) Complete removal of the remaining sacrificial layer and release of the freestanding structures.

Note that this specific architecture requires a final seed layer strip after the removal of the sacrificial mesa and critical point drying to avoid stiction. This eliminates the anti-stiction benefit of the vapour HF process, as the wafers can be wet-etched in a series of adequate etchants and finally critical-point dried. The vapour HF process is nevertheless demonstrated herein as it can be employed for different architectures and applications.

**Figure 11:**
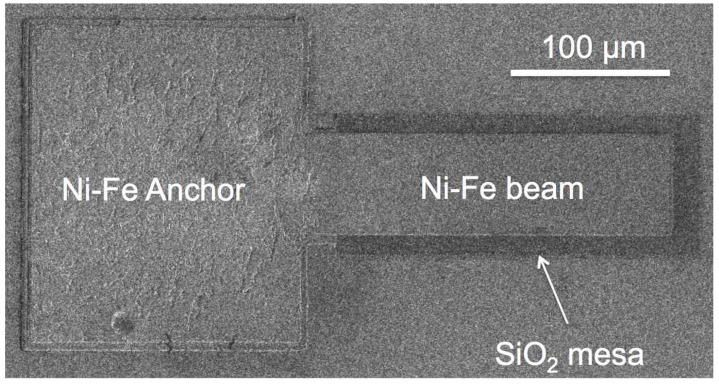
SEM image of a 200 µm long by 50 µm wide cantilever with the beam resting on the sacrificial silicon dioxide mesa.

Figure 11 shows a cantilever test structure with the beam resting on the silicon dioxide sacrificial mesa, corresponding to Figure 10g.

### 4.2. Release of the Cantilevers

The final step to produce freestanding cantilevers is the complete removal of the sacrificial layer by etching the remaining sacrificial oxide material with vapour HF (Figure 10h). The process was performed in a MEMSSTAR SVR-HF vapour phase dry release system. HF vapour is introduced in the process chamber at a flow rate of 150 sccm, and the pressure is ramped from 2 to 10 Torr in 1 to 2 min, and then kept constant for 300 s. The etch progress can be visualised by monitoring the generation of the by-products of the reaction

**Figure 12:**
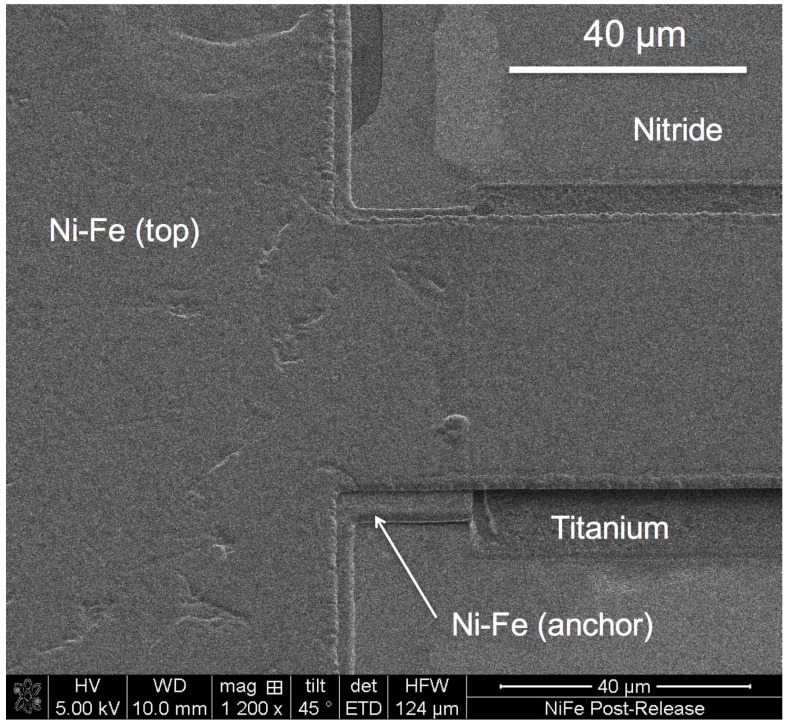
Ni-Fe cantilever beam after the removal of the sacrificial silicon dioxide mesa by vapour HF etch. The etched gap is clearly visible. The dark shadow under the cantilever beam is the titanium adhesion layer exposed after the removal of the sacrificial silicon oxide mesa.

SiO_2_ + 6HF ⟶ 2H^+^ + SiF_6_^2−^ + 2H_2_O (4)

using a built-in laser interferometer, indicating the reaction rate. The low temperature PECVD silicon dioxide facilitates a rapid process that releases the cantilevers in less than 300 s, without attacking the Ni-Fe structures or the structural silicon nitride. The complete release is verified by applying an external magnetic field and observing the magnetic cantilevers free to deflect and align their easy magnetisation axis (length) with the external magnetic field lines (see video in Supplementary Materials). Figure 12 shows a SEM image of the anchored end of a cantilever after the removal of the sacrificial SiO_2_ mesa.

**Figure 13:**
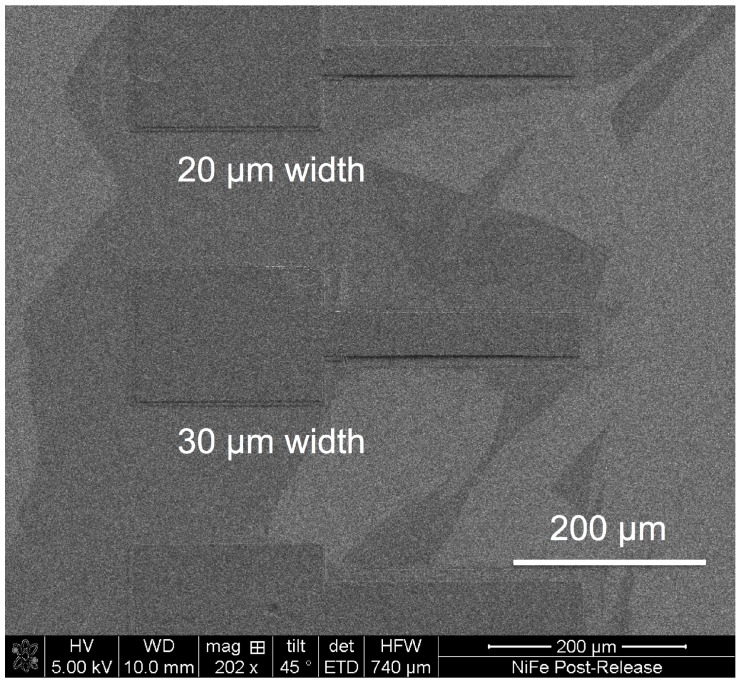
Released 200 µm long Ni-Fe cantilevers with widths of 20 and 30 µm.

The etch release is a room temperature process, which also ensures that no undesirable thermal loads are applied to the Ni-Fe, thereby ensuring that the residual stress is not increased. This can be visually verified by observing freestanding structures that are flat and free from curling effects, which indicates low stress gradient through the thickness of the film. Figure 13 shows 200 µm long cantilevers released with the described surface micromachining process.

**Figure 14:**
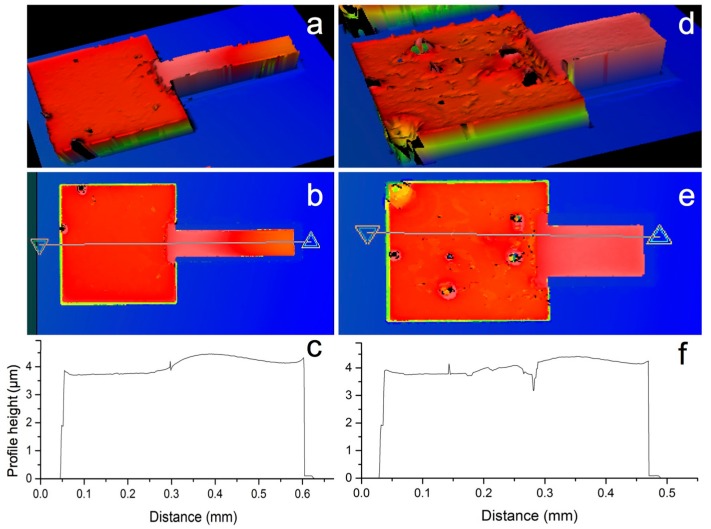
White light interferometry of two released cantilever beams: 300 µm long × 70 µm wide (**a**–**c**); and 200 µm long × 100 µm wide (**d**–**f**). 3D topology reconstructions (**a**,**d**); top views with the height measurement line (**b**,**e**); and measured profile height (**c**,**f**). Note the change in roughness between the pads (two electrodeposited layers stacked) and the beams (electrodeposited on a smooth sacrificial oxide mesa). Note that the surface roughness observed in (**d**) is due to the uneven growth of Ni-Fe, possibly associated to roughness of the underlying layer, current crowding effects due to topology defects, impurities, etc.

The SEM images show the complete removal of the sacrificial silicon dioxide, producing freestanding Ni-Fe cantilevers that, as a result of the improved processes detailed in this work, are not chemically attacked, nor do they suffer from excessive stress. Profiles were measured optically by white light interferometry to provide a measure of the curling of the beams and verify that they do not suffer from excessive stress induced by the processing. This is shown in Figure 14.

The vapour HF process is successfully proven as viable for use with ECD Ni-Fe, as no damage was observed on the ECD Ni-Fe and the PECVD silicon nitride. The full undercut width was also rapidly etched underneath the freestanding structures. No stiction effect was observed, no visible residue was left on the substrate, and no thermal load was applied.

## 5. Conclusions

A set of deposition and etching processes has been developed as a toolkit for the integration of surface micromachined ECD Ni-Fe in complex MEMS devices. The first process is a wet etch that uses a conventional ammonium persulfate chemistry, diluted in a sodium hydroxide solvent to enable the electrodeposition seed layer to be stripped with no detrimental effects on ECD Ni-Fe. Two PECVD processes are subsequently presented that enable the low-temperature deposition of structural silicon nitride and sacrificial silicon dioxide for use with ECD Ni-Fe. Finally, wet etch and vapour etch processes for the PECVD silicon dioxide are reported that enable the patterning of sacrificial structures and the complete removal of the sacrificial material, while preserving the structural PECVD silicon nitride.

All the reported processes avoid excessive thermal loads that are known to generate residual stress and stress gradients with consequent detrimental effects on Ni-Fe freestanding structures [25]. This is achieved by limiting the processing temperatures to below 200 °C, based on previously reported research [24].

The improved copper seed etch chemistry, the low-temperature PECVD sacrificial silicon dioxide and the corresponding wet etch patterning and vapour etch removal methods have been integrated into a process that emulates the manufacturing of complex magnetic MEMS devices. It is worth noting that the developed processes can be used in any combination and for a range of different applications. An all-wet surface micromachining process can be used, for instance, for the two-step cantilever architecture shown, since critical point drying is needed after the final seed etch step. Conversely, if a standard Ni-Fe beam or membrane is fabricated with no via-anchor structure (no seed layer underneath the sacrificial material), the vapour HF process can be used to avoid stiction without the need for critical point drying.

The reported results represent therefore a useful reference for the manufacturing of complex MEMS devices with integrated ECD Ni-Fe elements.

## Figures and Tables

**Table 1:** Chemical composition of the Ni-Fe electroplating bath.

Component	Concentration (gL^−1^)
NiCl_2_·6H_2_O	110
FeCl_2_·4H_2_O	8
H_3_BO_3_	25
Saccharin	1
Na dodecyl sulphate	0.1
HCl	5

**Table 2:** Chemical composition of the improved copper etching solution.

Component	Quantity
(NH_4_)_2_S_2_O_8_	50 gL^−1^
C_6_H_8_O_7_	50 gL^−1^
NaOH	1 M

**Table 3:** Process parameters for the PECVD deposition of 2 µm of sacrificial SiO_2_.

Process Parameter	Value
SiH_4_ flow rate	6 sccm
N_2_O flow rate	1420 sccm
RF frequency	380 kHz
RF power	60 W
Wafer holder temperature	120 °C
Gas inlet unit temperature	120 °C
Chamber pressure	550 mTorr
Deposition time	53 min

**Table 4:** Process parameters for the PECVD deposition of 200 nm of structural Si_3_N_4_.

Process Parameter	Value
N_2_ flow rate	1900 sccm
SiH_4_ flow rate	120 sccm
NH_3_ flow rate	8 sccm
RF frequency	13.56 MHz
RF power	60 W
Wafer holder temperature	200 °C
Gas inlet unit temperature	200 °C
Chamber pressure	550 mTorr
Deposition time	7.5 min

**Table 5:** Wet-Etch Rates for the PECVD SiO_2_ and Si_3_N_4_.

Etchant	Material	Approximate Etch Rate
1:2 [BOE 10:1] in DI water	PECVD SiO_2_	1500 nm/min
1:2 [BOE 10:1] in DI water	PECVD Si_3_N_4_	20 nm/min
1:10 [BOE 10:1] in DI water	PECVD SiO_2_	160 nm/min
1:10 [BOE 10:1] in DI water	PECVD Si_3_N_4_	not detected

## Supplementary Materials

The following are available online at www.mdpi.com/1996-1944/10/3/323/s1.
